# Supplementary figures and images for: A conserved fungal Knr4/Smi1 protein is crucial for maintaining cell wall stress tolerance and host plant pathogenesis
Source: PLoS Pathog. 2025 Jan 9;21(1):e1012769. doi: 10.1371/journal.ppat.1012769 (PMC11717356; doi:10.1371/journal.ppat.1012769)

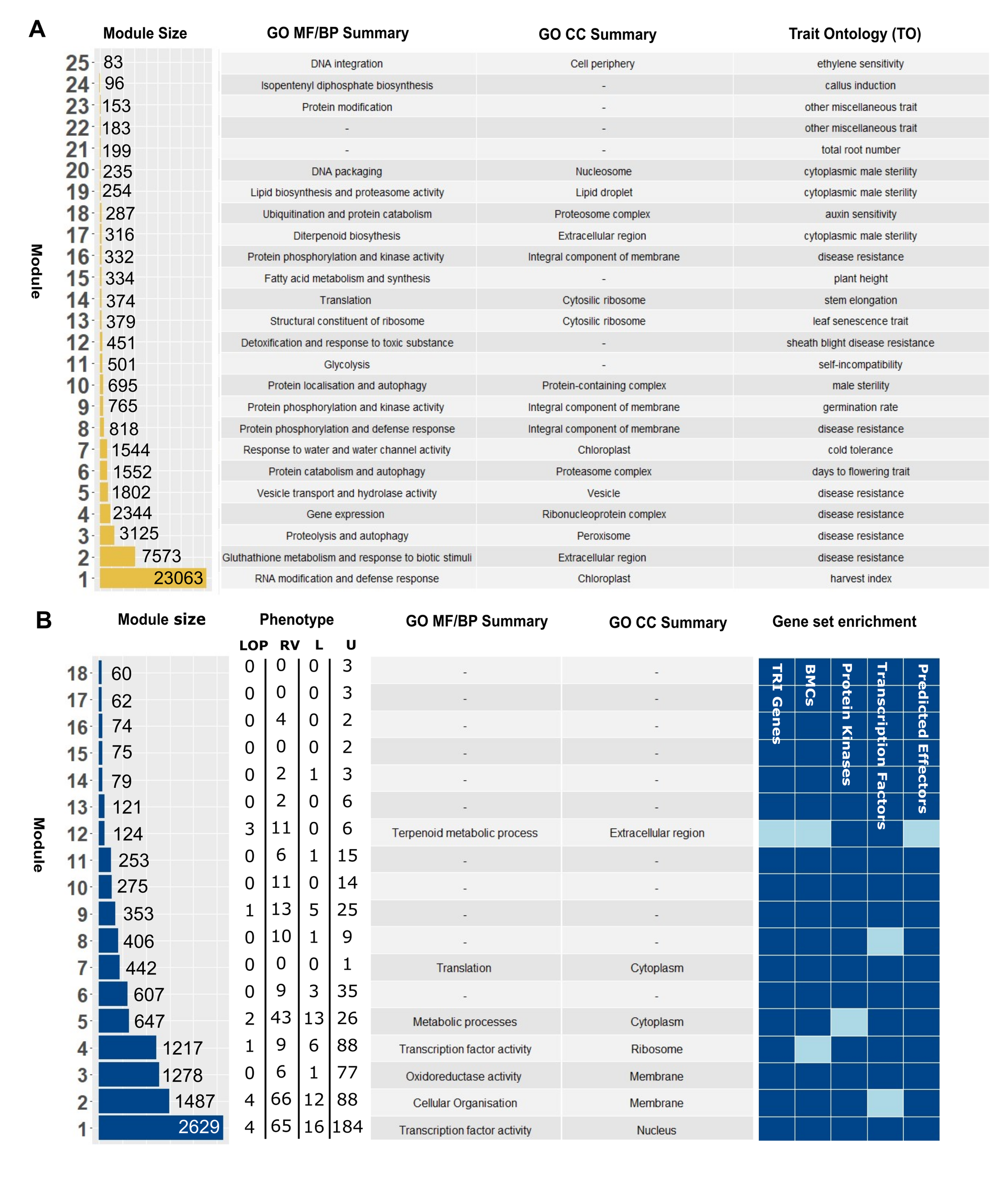

Supplement: S1 Fig — A. Summary of all modules in the wheat network, including module size (number of genes), Gene Ontology (GO) and Trait Ontology (TO) enrichment summaries. B. Summary of all modules in the fungal network, including modules size, Gene Ontology (GO) enrichment summaries and Gene Set Enrichment Analysis (GSEA). Light blue indicates significant enrichment (p < 0.05) for the given term. The number of genes with different phenotypes in PHI-base are depicted, with LOP, RV, L and U denoting different PHI-base phenotypes (LOP = Loss of pathogenicity; RV = Reduced virulence; L = Lethal; U = Unaffected pathogenicity) (Urban et al., 2022) [95]. (TIF) [file ppat.1012769.s001.tif]

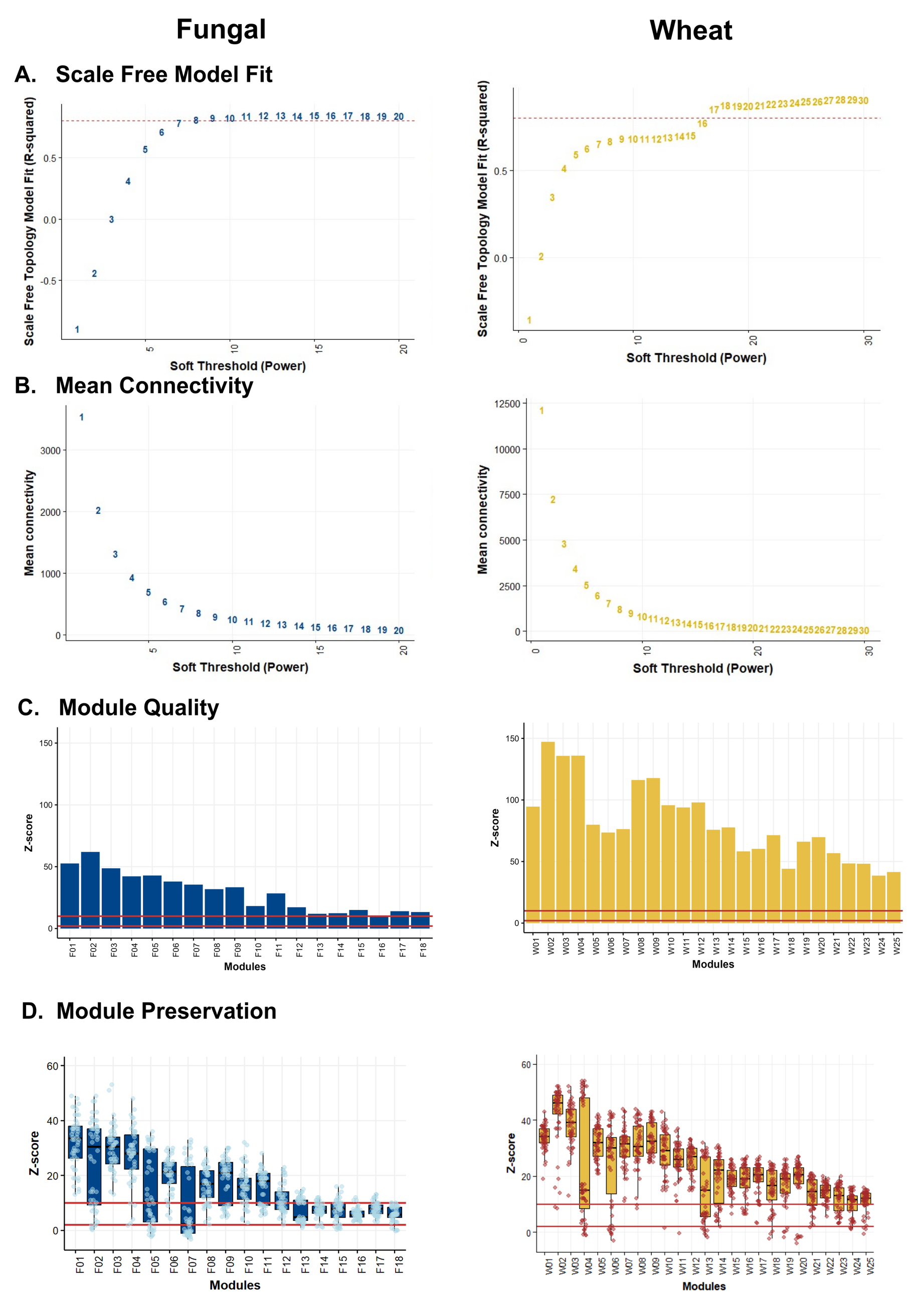

Supplement: S2 Fig — A. Strength of correlation of network model (R-squared value) to scale free model at different soft thresholding powers. Dotted red line is at an R-squared value of 0.80, the threshold needed for generating a WGCNA network. B. Mean connectivity of genes in each network at different soft thresholding powers. A low mean connectivity is desired to meet the scale free network criteria. C. Module quality across all modules as determined by a Z-score calculation. Solid red lines at minimum quality (Z = 2) and high quality scores (Z = 10). D. Module preservation as determined by Z-score calculation against 50 random test networks. Solid red lines at minimum preservation (Z = 2) and high preservation scores (Z = 10). (TIF) [file ppat.1012769.s002.tif]

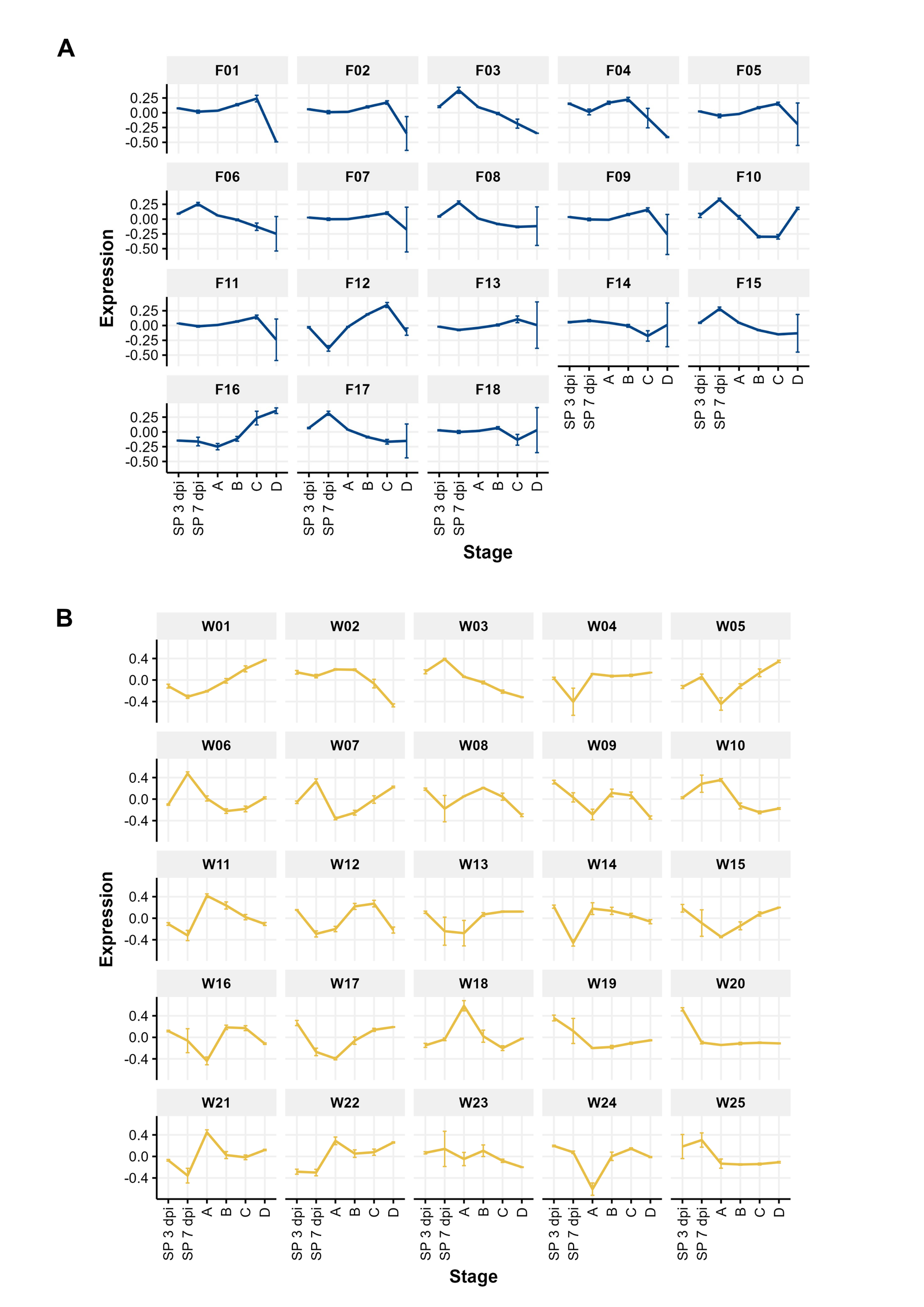

Supplement: S3 Fig — Eigengene summarised expression plots illustrating the expression patterns of genes in A Fungal and B Wheat modules across different stages namely spikelet tissue (SP) at 3 and 7 dpi and A = Late symptomatic, B = Early symptomatic, C = Late symptomless, and D = Early symptomless. (TIF) [file ppat.1012769.s003.tif]

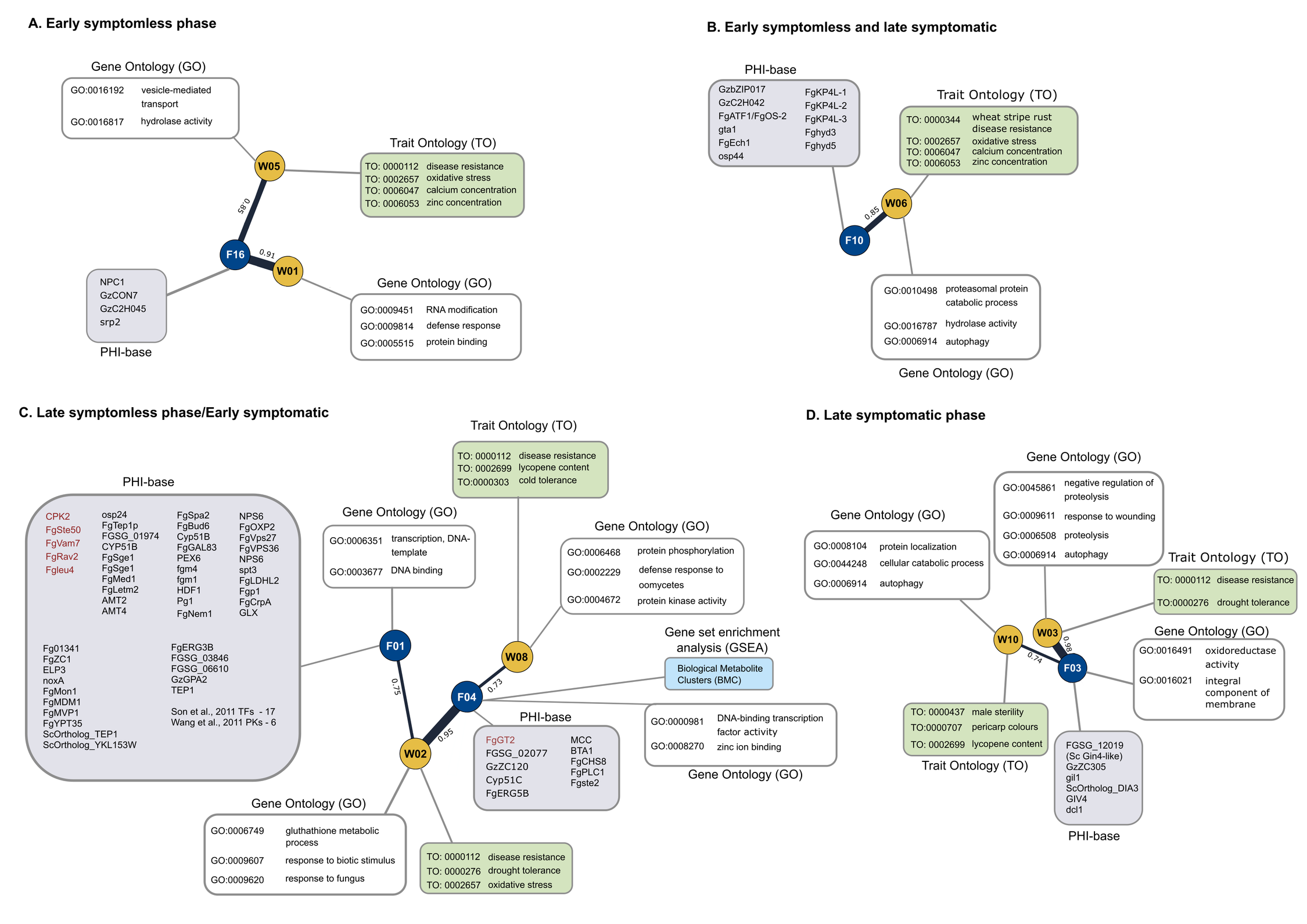

Supplement: S4 Fig — Fungal modules (F) and wheat modules (W) depicted with significant enrichment annotations. Fungal modules are additionally annotated with known phenotypes from PHI-base. Genes listed in red in the PHI-base annotation (grey box) exhibit a loss of pathogenicity phenotype when deleted, while the remaining genes display a reduced virulence phenotype when deleted. Plots are separated by modules with highest expression in a given stage of infection, namely A. Early symptomless, B. Early symptomless and late symptomatic, C. Late symptomless/Early symptomatic, and D. Late symptomatic. (TIF) [file ppat.1012769.s004.tif]

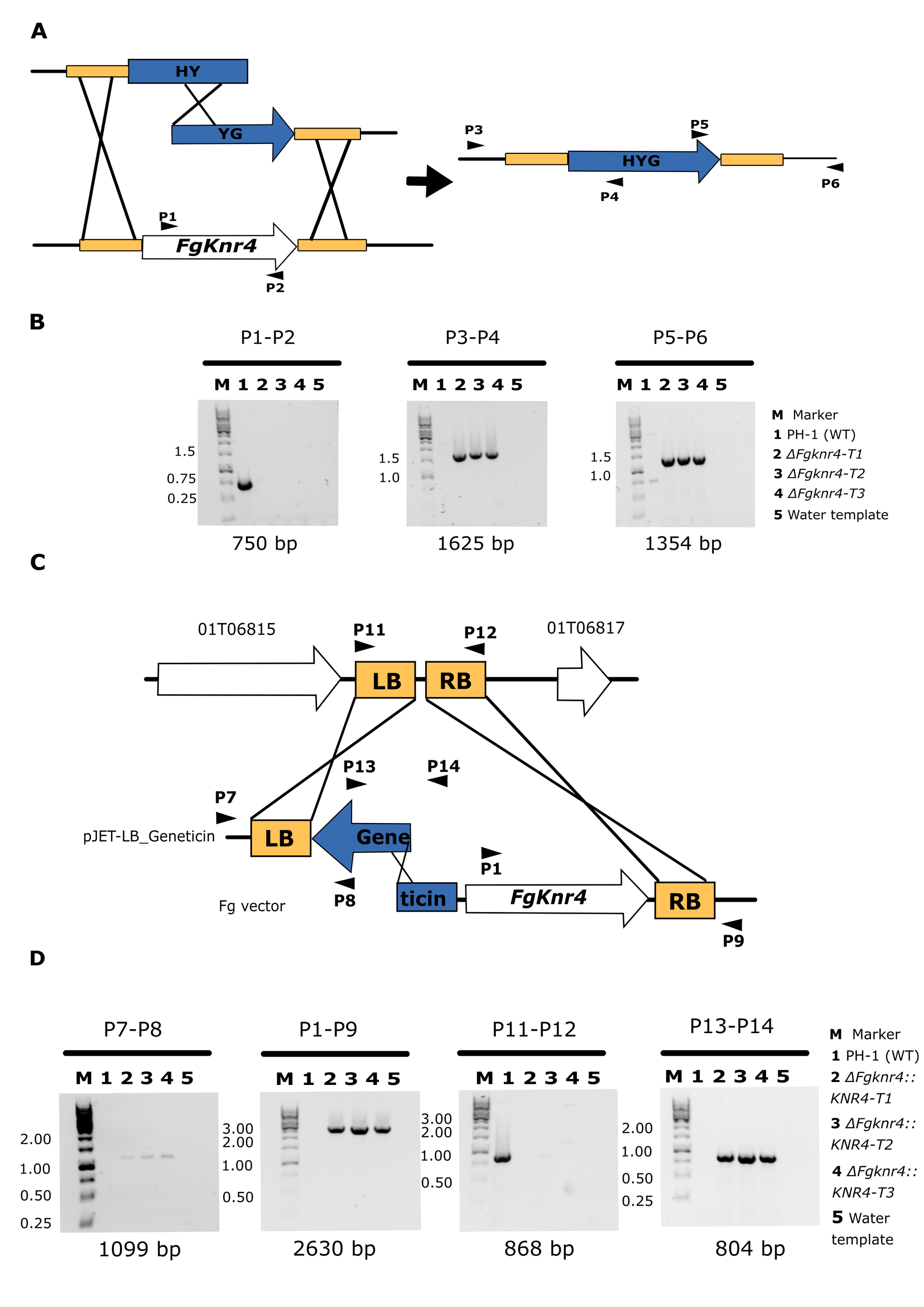

Supplement: S5 Fig — A. Schematic for the hygromycin split marker deletion strategy including diagnostic primer locations (P1-6). B. Diagnostic PCR with primer sets depicted in panel A. PCR samples were separated on 0.75% agarose gel with a 1 kb DNA ladder. The expected amplicon size is given below the corresponding gel image. C. Schematic of gene complementation into the Fg transformation locus (Darino et al. 2024) [128], including diagnostic primer locations. D. Diagnostic PCR with primer sets depicted in panel C. PCR samples were separated on 0.75% agarose gel with a 1 kb DNA ladder. Expected amplicon sizes are written below the corresponding gel image. (TIF) [file ppat.1012769.s005.tif]

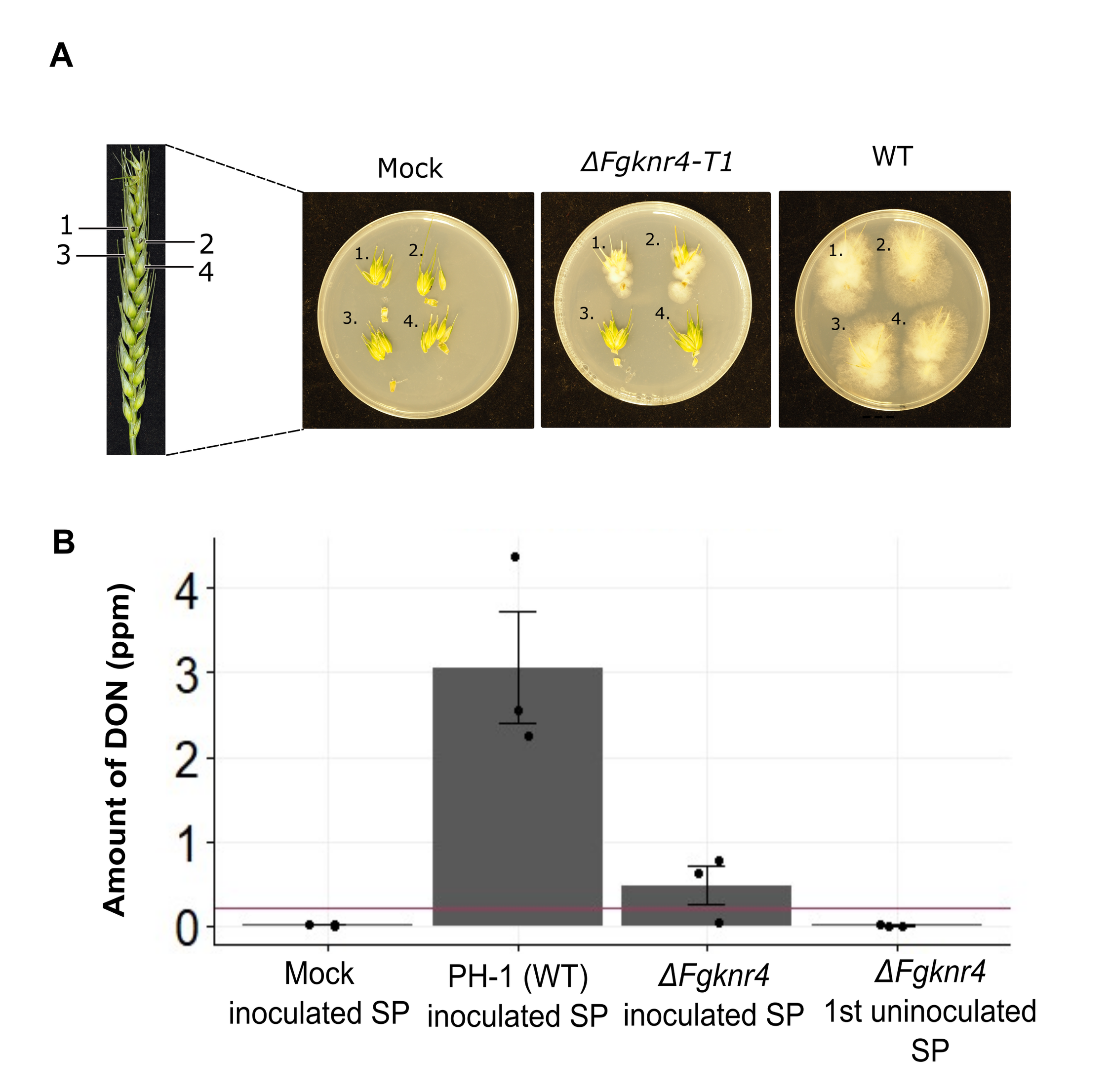

Supplement: S6 Fig — A. Dissection of wheat spikes followed by separation of infected wheat spikelet and rachis tissues and subsequent plating onto synthetic nutrient agar (SNA) separated at 15 dpi. Plate images taken 3 days later. B. ELISA based quantification of DON in dissected spikelet (SP) samples of wheat inoculated with wild-type (PH-1) and ΔFgknr4 at 15 dpi. Quantity of DON is measured in parts per million (ppm) and the detection threshold of the kit is indicated by a red line (ppm = 0.2). This experiment was replicated using tissue from three separate wheat spikes (N = 3). (TIF) [file ppat.1012769.s006.tif]

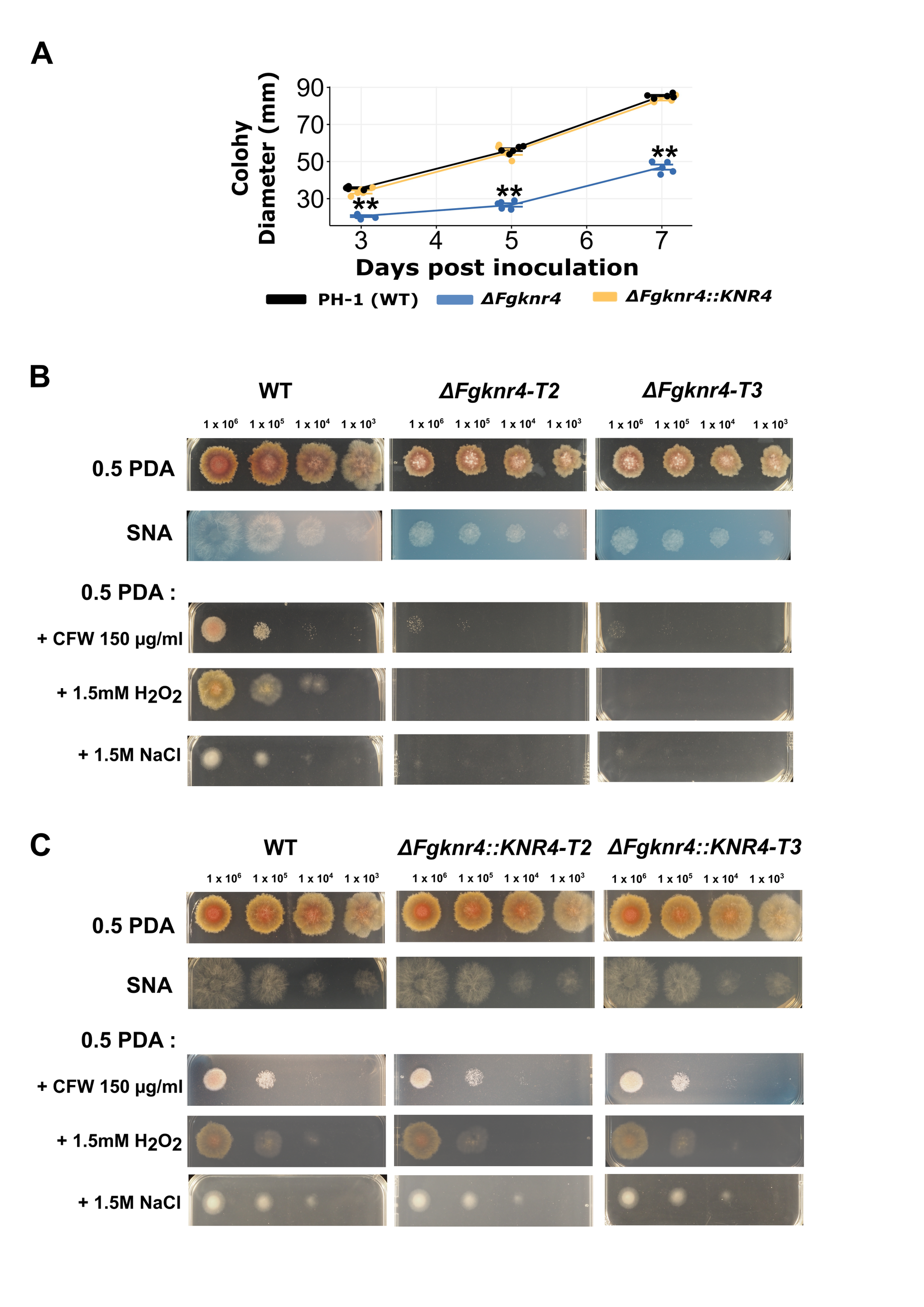

Supplement: S7 Fig — A. Mean colony diameter of wild-type (WT), ΔFgknr4, and ΔFgknr4::KNR4 grown on Potato Dextrose Agar (PDA) (N = 5). B. Dilution series of WT and additional ΔFgknr4 transformants (T2 and T3) on Synthetic Nutrient Agar (SNA) and half-strength Potato Dextrose Agar (0.5 PDA) with and without the addition of single stresses. C. Dilution series of WT and additional ΔFgknr4::KNR4 transformants (T2 and T3) on Synthetic Nutrient Agar (SNA) and half-strength Potato Dextrose Agar (0.5 PDA) with and without the addition of single stresses. The dilution series begins at 1 x 10^6^ and continues with 10-fold dilutions. Images were taken after 3 days and these experiments were replicated twice with similar results. (TIF) [file ppat.1012769.s007.tif]

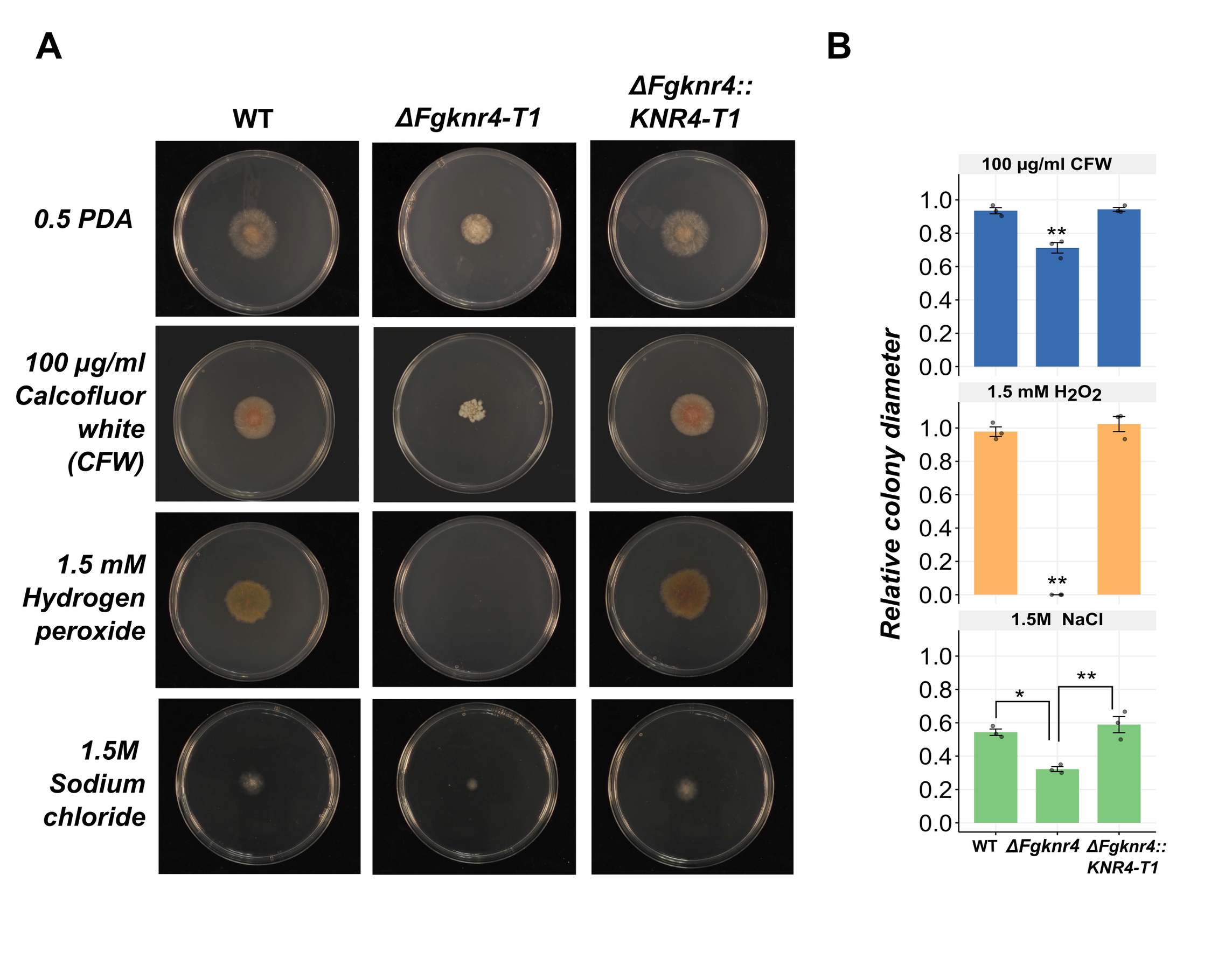

Supplement: S8 Fig — A. Wild-type (WT), ΔFgknr4, and ΔFgknr4::KNR4 grown on half-strength Potato Dextrose Agar (0.5 PDA) with and without the addition of single stresses. Plates were spotted with 20 μl of 1 x 10^6^ conidia/ml conidial suspensions. Images were taken after 3 days. B. Colony diameters of WT, ΔFgknr4, and ΔFgknr4::KNR4 grown on 0.5 PDA supplemented with calcofluor white (CFW), hydrogen peroxide (H2O2), and sodium chloride (NaCl) relative to growth on 0.5 PDA without stress. Significance is denoted as * = p ≤ 0.05 and ** = p ≤ 0.01. Significance was determined by a one-way ANOVA followed by Tukey HSD correction. (TIF) [file ppat.1012769.s008.tif]

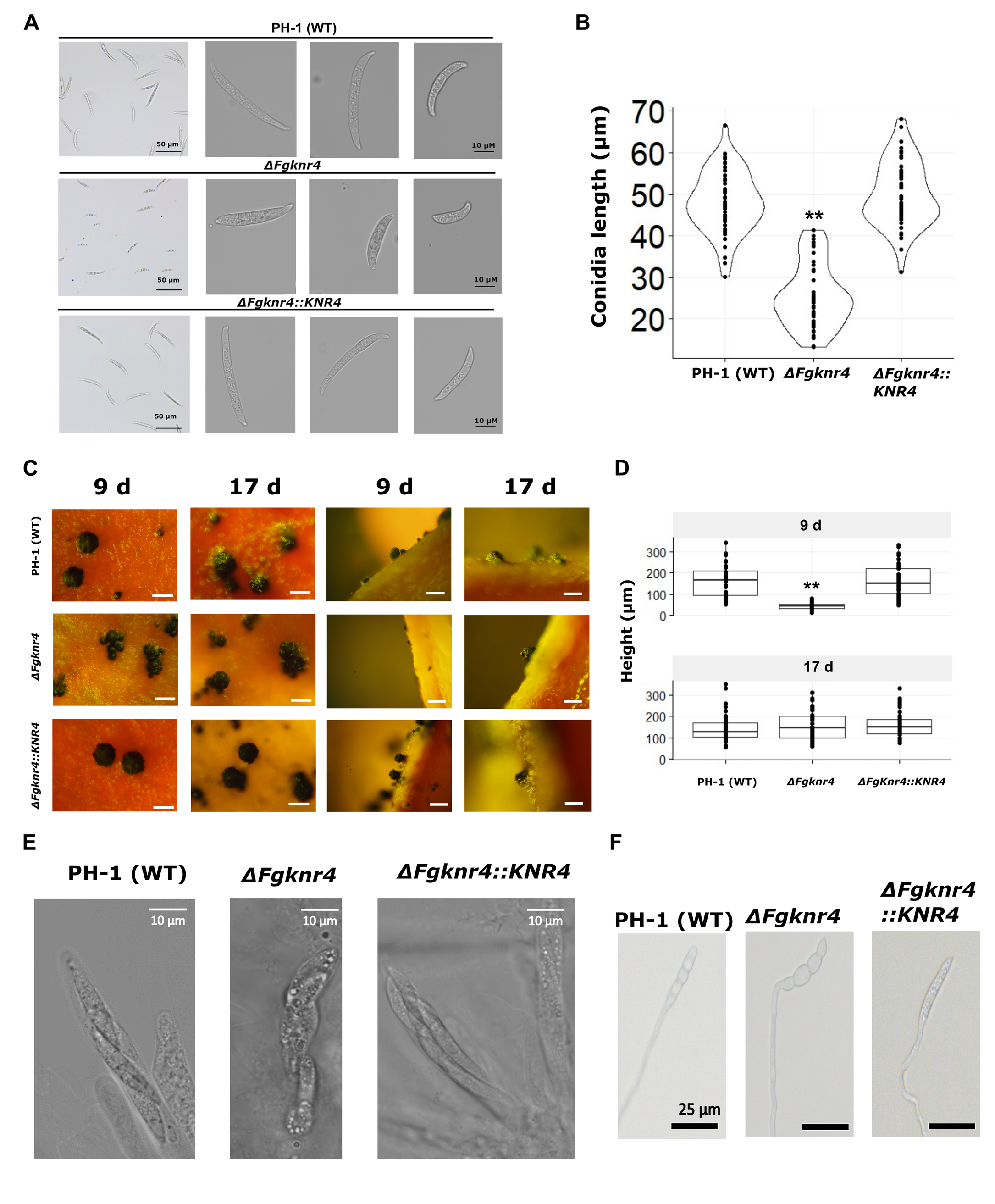

Supplement: S9 Fig — A. Decreased conidial length observed in ΔFgknr4. Single conidial images to represent long, middle length, and short conidia across strains. B. Distribution of conidial length from N = 50 for WT, ΔFgknr4, and ΔFgknr4::KNR4 strains. C. Representative perithecia images taken after perithecia induction in carrot agar medium for WT, ΔFgknr4, and ΔFgknr4::KNR4 strains. Images taken from above (left panels) and from agar sections placed on slides (right panels) on day 9 and day 17. Scale bar = 500 μm. D. Mean perithecia height of WT, ΔFgknr4, and ΔFgknr4::KNR4 after 9 and 17 days (N = 40). E. Ascospores in intact ascus produced by wild-type (WT), ΔFgknr4 or ΔFgknr4::KNR4 strains. Scale bar = 10 μM. F. Ascospores obtained from squashed perithecia of wild-type (WT), ΔFgknr4 or ΔFgknr4::KNR4 strains are viable and form germ tubes. Scale bar = 25 μm. Significance is denoted as ** = p ≤ 0.01. Significance was determined by a one-way ANOVA followed by Tukey HSD correction. (TIF) [file ppat.1012769.s009.tif]

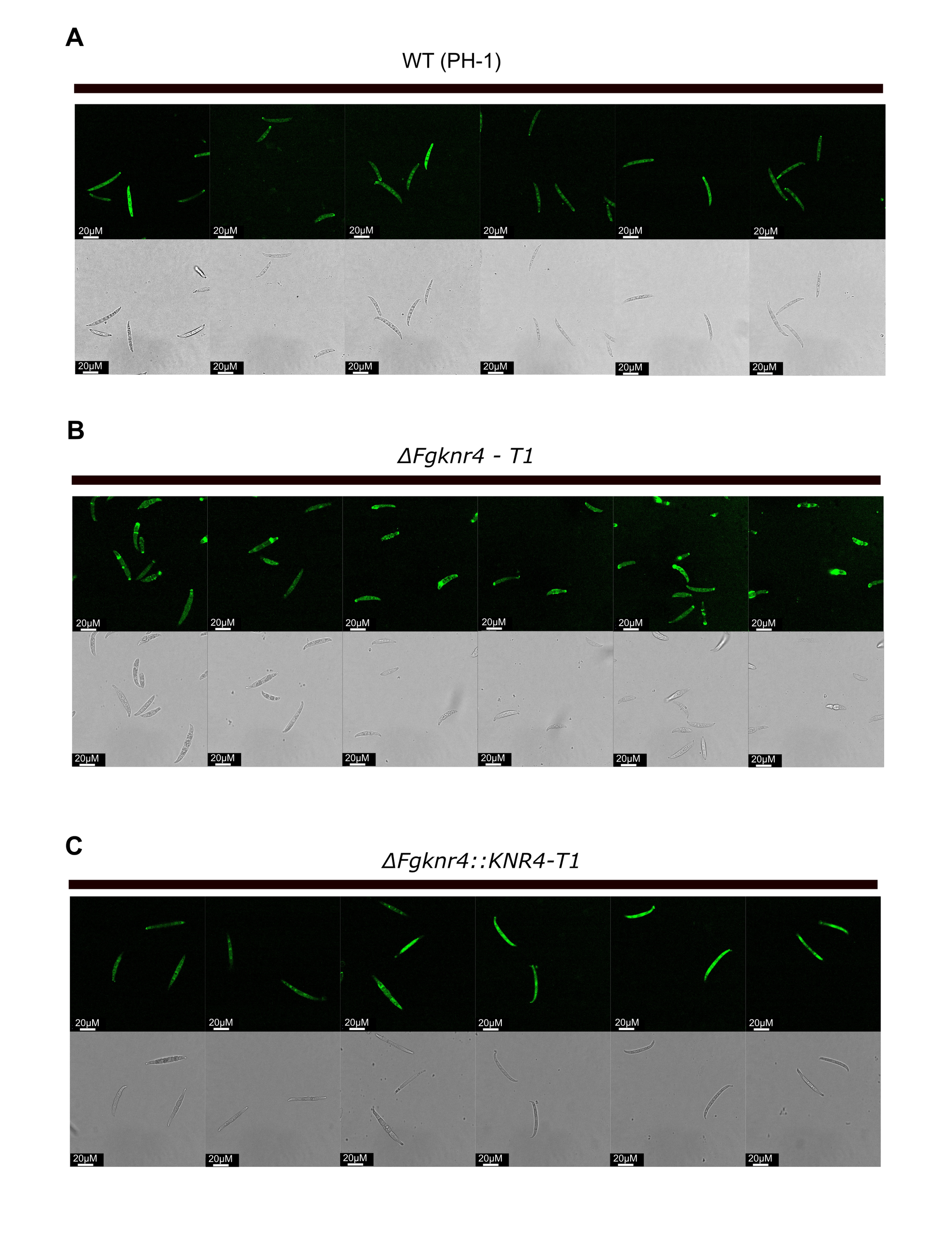

Supplement: S10 Fig — Visualisation of chitin-stained conidia with Wheat Germ Agglutinin Alexa Fluor 488 Conjugate (WGA) in wild-type (WT) (A), ΔFgknr4 (B) and ΔFgknr4::KNR4 (C). (TIF) [file ppat.1012769.s010.tif]

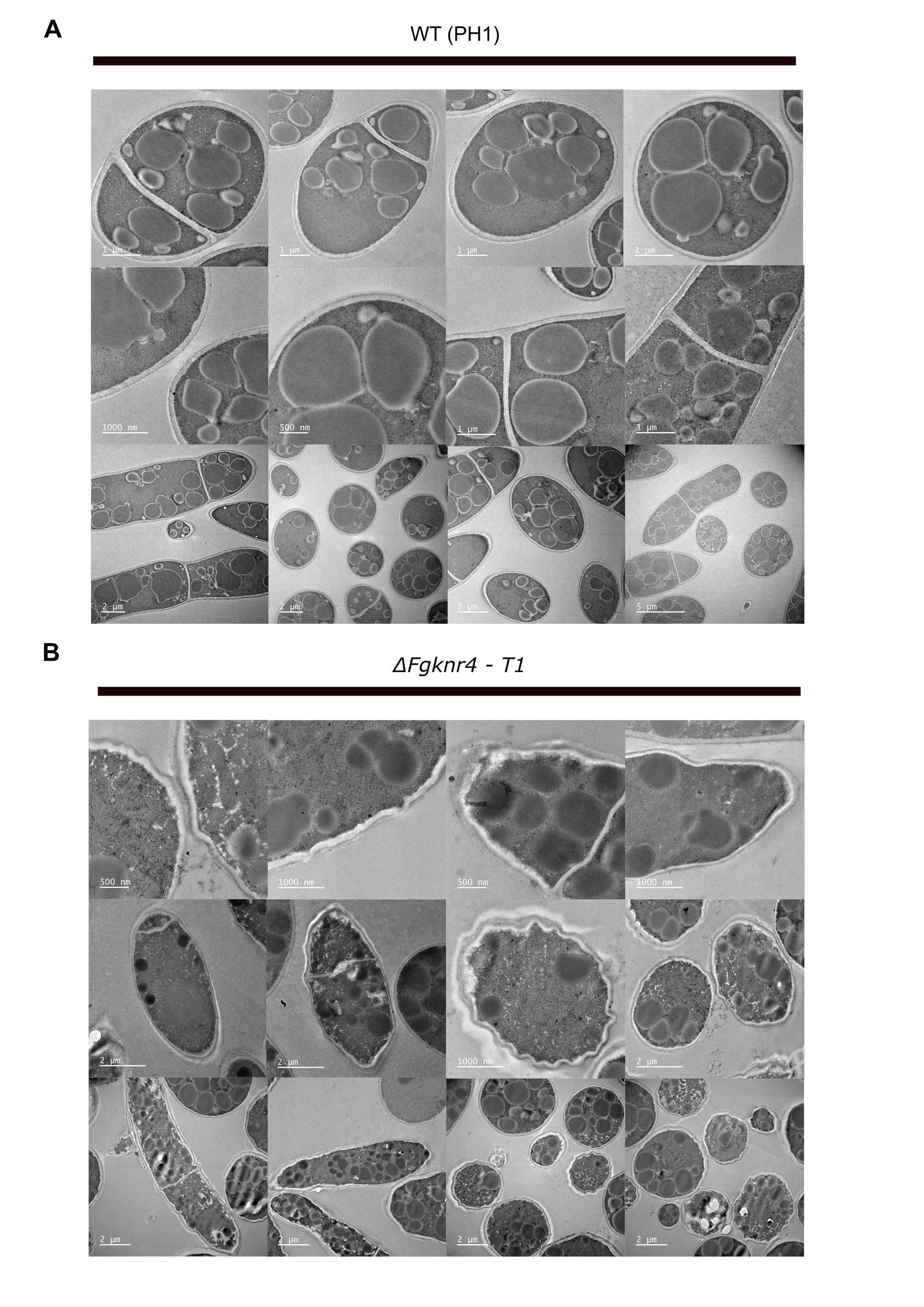

Supplement: S11 Fig — TEM imaging of wild-type (A) and ΔFgknr4 (B) conidia, showing differences in cell wall structure and different magnifications. (TIF) [file ppat.1012769.s011.tif]

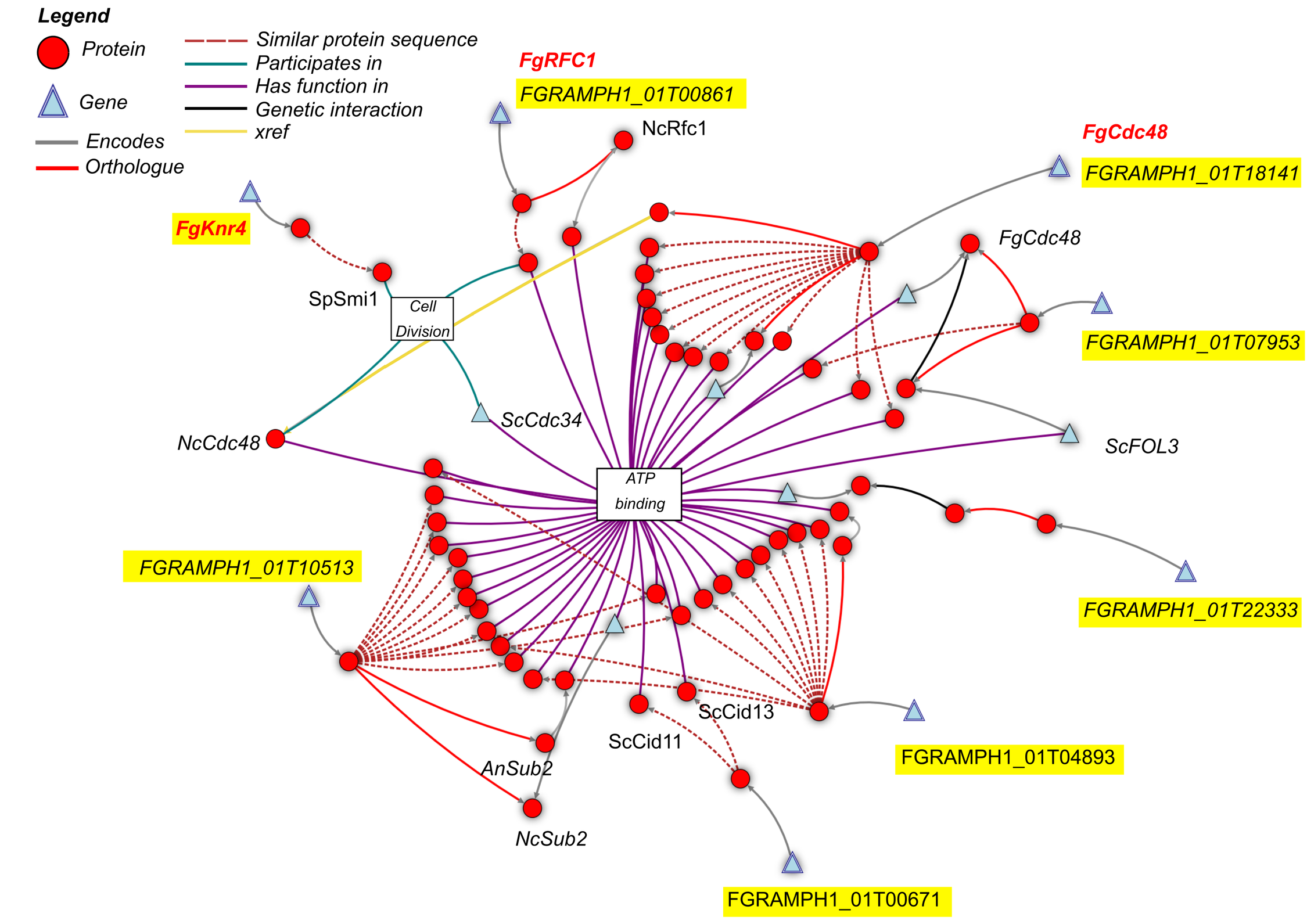

Supplement: S12 Fig — Knowledge network demonstrating shared relationships between eight of the 15 candidate genes from module F16. Genes belonging to the candidate gene list are highlighted in yellow. (TIF) [file ppat.1012769.s012.tif]

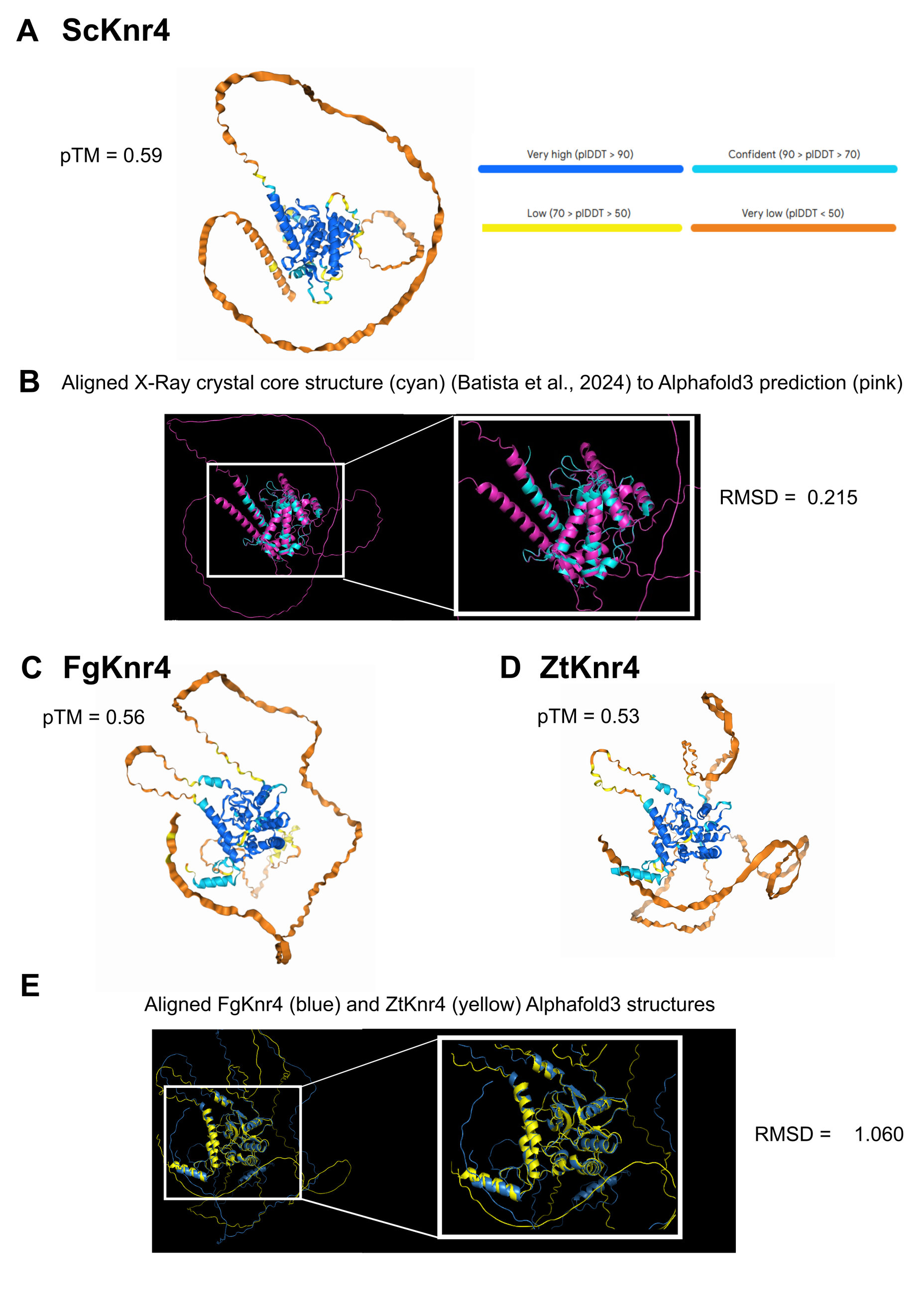

Supplement: S13 Fig — A. Alphafold3 model of ScKnr4 structure, with residues colours corresponding to pIDDT confidence scores. The predicted template modelling (TM) score is provided next to the structure. B. Validation of ScKnr4 Alphafold3 prediction (pink) through alignment with crystal structure of ScKnr4 core (blue). C. FgKnr4 Alphafold3 predicted folded structure, with residue colours corresponding to pIDDT confidence scores and displayed with associated pTM score. D. ZtKnr4 Alphafold3 predicted folded structure, with residues colours corresponding to pIDDT confidence scores and displayed with associated pTM score. E. The alignment of the predicted structures of FgKnr4 (blue) and ZtKnr4 (yellow). (TIF) [file ppat.1012769.s013.tif]

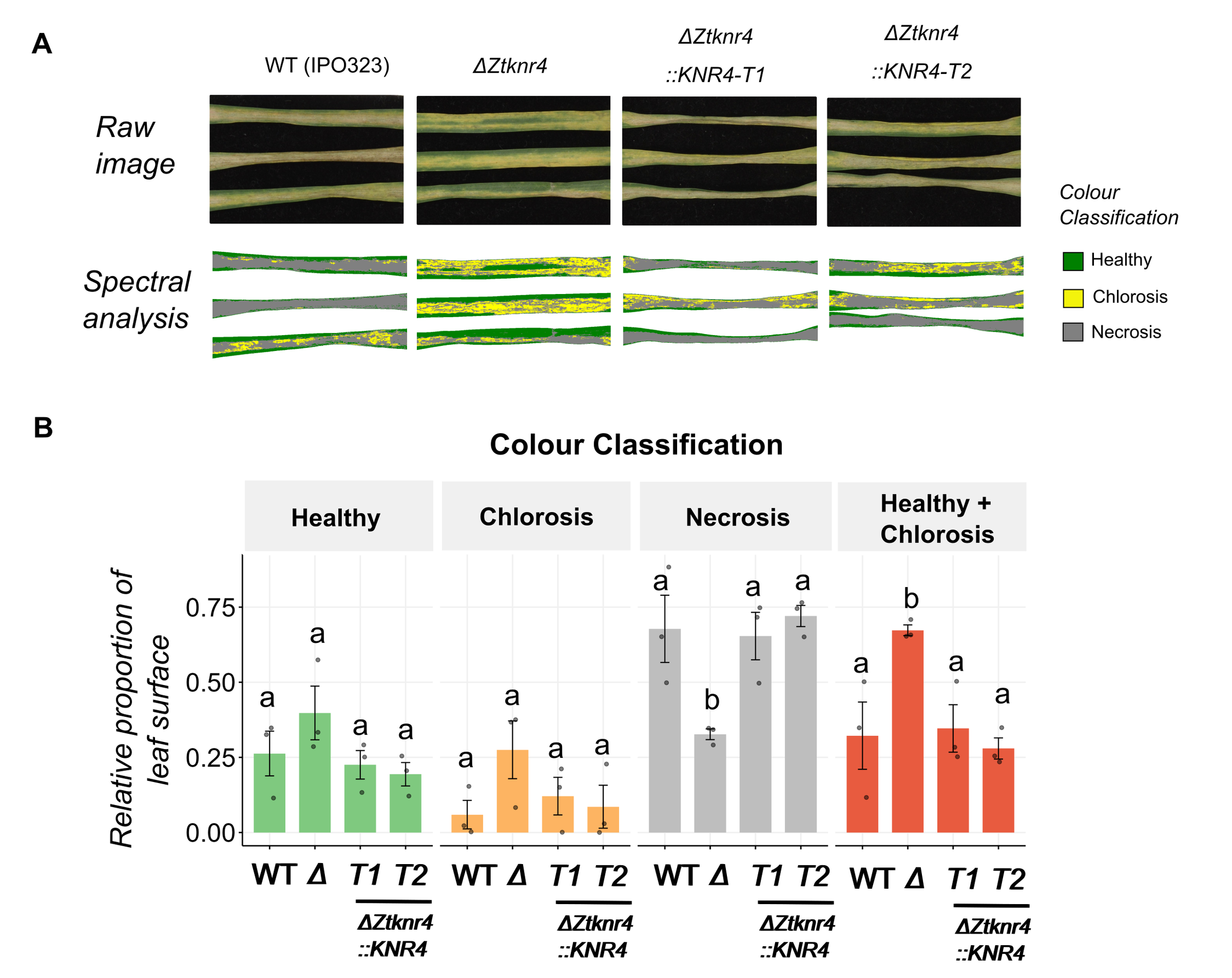

Supplement: S14 Fig — A. Upper panel includes raw images of detached wheat leaves inoculated with wild-type (WT) Z. tritici (IPO323), ΔZtknr4 mutant strain, and two complemented strains (ΔZtknr4::KNR4-T1 and T2) as presented in Fig 9. Images taken at 20 dpi. Lower panel depicts the colour classification of tissue exhibiting healthy (green), chlorosis (yellow), and necrosis (grey) phenotypes as determined by LemnaGrid image analysis system. B. Lower panel shows the relative proportion of each leaf surface classified as healthy, chlorosis, or necrosis phenotypes using LemnaGrid image analysis system (N = 3). Significance was determined by a one-way ANOVA followed by a Tukey HSD correction (p ≤ 0.05). (TIF) [file ppat.1012769.s014.tif]

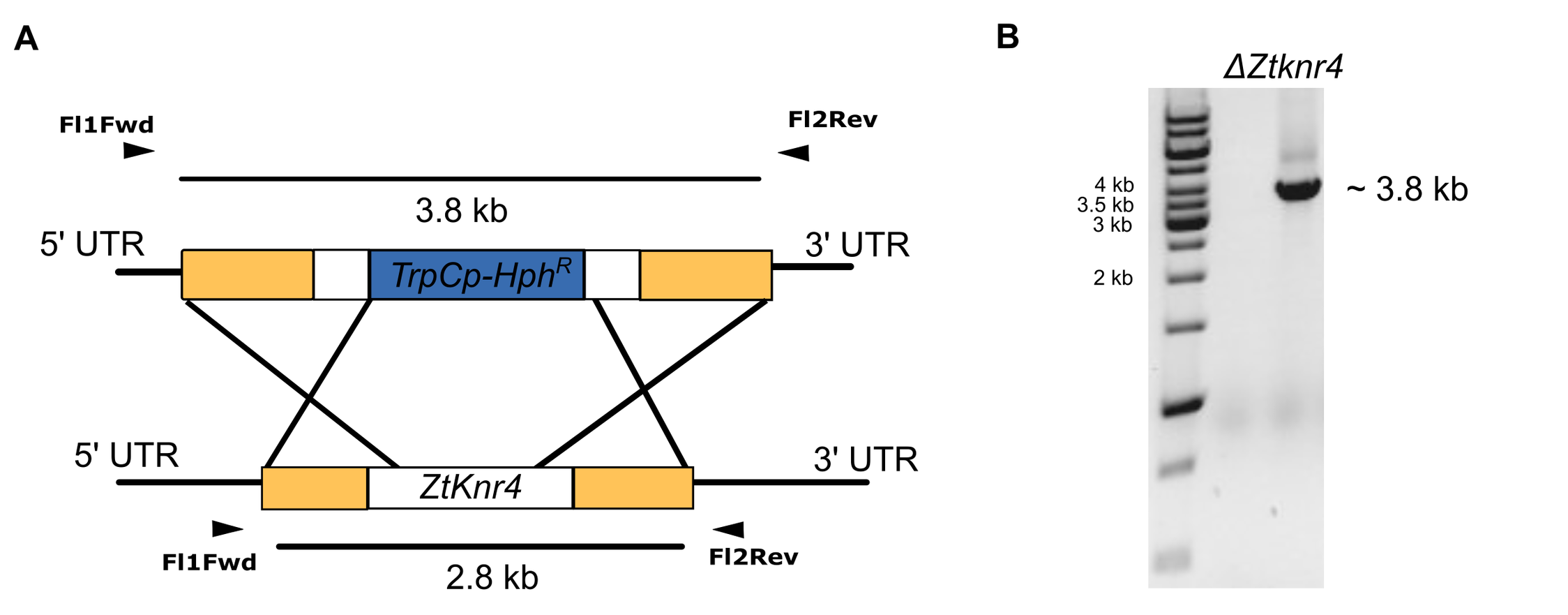

Supplement: S15 Fig — A. Hygromycin (HphR) replacement cassette inserts at ZtKnr4 locus through homologous recombination via homologous flanks (yellow and white). B. Diagnostic PCR demonstrating presence of large insertion fragment in ΔZtknr4 transformant using Fl1Fwd and Fl2Rev primers. (TIF) [file ppat.1012769.s015.tif]
